# Learning modulation of odor representations: new findings from *Arc*-indexed networks

**DOI:** 10.3389/fncel.2014.00423

**Published:** 2014-12-19

**Authors:** Qi Yuan, Carolyn W. Harley

**Affiliations:** ^1^Division of Biomedical Sciences, Faculty of Medicine, Memorial University of NewfoundlandSt. John’s, NL, Canada; ^2^Department of Psychology, Faculty of Science, Memorial University of NewfoundlandSt. John’s, NL, Canada

**Keywords:** anterior piriform cortex, olfactory bulb, early odor preference learning, odor discrimination learning, *Arc*, viability, sparse coding

## Abstract

We first review our understanding of odor representations in rodent olfactory bulb (OB) and anterior piriform cortex (APC). We then consider learning-induced representation changes. Finally we describe the perspective on network representations gained from examining *Arc*-indexed odor networks of awake rats.* Arc*-indexed networks are sparse and distributed, consistent with current views. However *Arc* provides representations of repeated odors. *Arc*-indexed repeated odor representations are quite variable. Sparse representations are assumed to be compact and reliable memory codes. *Arc* suggests this is not necessarily the case. The variability seen is consistent with electrophysiology in awake animals and may reflect top-down cortical modulation of context. *Arc*-indexing shows that distinct odors share larger than predicted neuron pools. These may be low-threshold neuronal subsets. Learning’s effect on *Arc*-indexed representations is to increase the stable or overlapping component of rewarded odor representations. This component can decrease for similar odors when their discrimination is rewarded. The learning effects seen are supported by electrophysiology, but mechanisms remain to be elucidated.

Here we first characterize our understanding of odor representations in the olfactory bulb (OB) and anterior piriform cortex (APC). We next review learning-related modulation of those odor representations. Finally, we discuss data using *Arc*-indexed odor representations. These data modify our view of odor representations and their modulation by reward. How *Arc* expression is recruited by odor is also considered.

## Odor representations in the olfactory bulb and anterior piriform cortex

It has long been recognized that odor representations require across-fiber coding (Chaput and Holley, 1985). This means that they are population or network representations from the beginning. Olfactory sensory neurons encode molecular features of which there is a large variety (Mombaerts et al., 1996; Mori et al., 2006; Saito et al., 2009). Natural odors activate multiple sensory neurons (Lin da et al., 2006; Mori et al., 2006).

**Figure 1:**
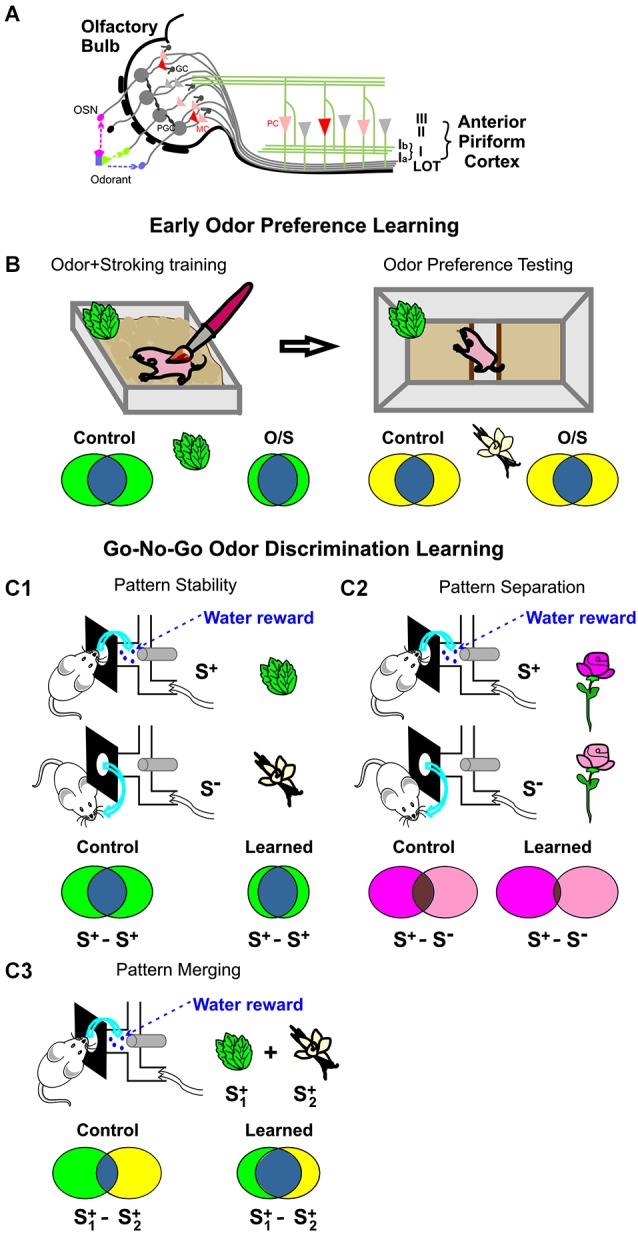
**Odor network representations in early olfactory cortices. (A)** Simplified olfactory bulb and anterior piriform cortex (APC) circuitry. Odorant molecular features are detected by olfactory sensory neurons (OSNs) in the nose and transmitted to the glomeruli of the olfactory bulb where OSNs synapse with output mitral cells (MCs). Mitral cells project to multiple pyramidal cells (PCs) in the APC *via* the lateral olfactory tract (LOT). Mitral cell output is regulated by interneurons at both the glomerular layer (periglomerular cells, PGCs) and the granule cell layer (granule cells, GCs). Piriform PCs receive convergent MC inputs and respond to individual odors. **(B)** *Arc*-indexed representation for early odor preference learning. A week-old rat pup undergoes odor+stroking (O/S) training with peppermint-scented bedding. This O/S training results in a preference for peppermint-scented bedding when the pup is tested 24 h later. *Arc* visualization to repeated peppermint following training reveals increased proportions of reliably activated neurons. Increased reliability is not seen when the pup is exposed to a control odor vanillin (Shakhawat et al., 2014a). **(C1–C3)** *Arc*-indexed representation of adult rat “go-no-go” odor discrimination learning in the APC. **(C1)** Odor reward learning increases the stability of the rewarded odor representation. S^+^ refers to positive rewarded odor stimulus (e.g., peppermint); S^−^ refers to negative unrewarded odor stimulus (e.g., vanillin; Shakhawat et al., 2014b). When a trained rat is exposed to S^+^, the proportion of neurons recruiting *Arc* twice is increased. **(C2)** Discrimination of highly similar odors leads to pattern separation. There is less overlap between the two similar odor representations after discrimination learning than before. **(C3)** Reward training with an odor mixture increases representational overlap between the two component odors. ${\text{S}}_{\text{1}}^{\text{+}}$ and ${\text{S}}_{\text{2}}^{\text{+}}$ refer to the components of the rewarded odor mixture. A third odor is used as non-rewarded odor (not shown).

Laurent (1997) highlighted the importance of distinguishing between maps (circuitry) and spatiotemporal codes for odors. He referred to the apparent chemotopic spatial organization of glomerular odor input which has been widely accepted (Rubin and Katz, 1999; Xu et al., 2000; Wachowiak and Cohen, 2001; Leon and Johnson, 2003; Soucy et al., 2009; see Figure 1A). But recent imaging evidence with single glomerular resolution (Ma et al., 2012) and theoretical analyses (Cleland, 2010) argue for a lack of chemotopic mapping at the glomerular level (see also Lin da et al., 2006). Thus odor representations are distributed representations even at the glomerular level as chemical characteristics do not predict odor maps. Nonetheless structurally-related odors activate similar distributed networks (Ma et al., 2012).

For mitral cells in the OB, an assumption that odor representations were dense and spatially specific has also evolved. A dramatic change in our understanding of mitral cell representations occurred when recordings were compared in anesthetized and awake mice (Rinberg et al., 2006). Under anesthesia, responses are driven by sensory input and occur against low spontaneous firing similar to antennal projection neurons in invertebrates (e.g., Krofczik et al., 2008). When awake, spontaneous activity is high (~20 Hz), and response to odor is weak and variable (Rinberg et al., 2006; Doucette and Restrepo, 2008; Zhan and Luo, 2010). Neuromodulatory input (Rinberg et al., 2006; Mandairon and Linster, 2009; Doucette et al., 2011), context (Kay and Laurent, 1999; Doucette and Restrepo, 2008; Mandairon et al., 2014), and other cortical top-down (Chapuis et al., 2013; Rothermel and Wachowiak, 2014) influences play a role in these awake representations. Odor decoding must depend on stable and/or synchronized elements within the population. Granule cells have been less studied, but evidence suggests they also have odor encoding features (Busto et al., 2009).

The distributed and sparse network representations seen in OB also occur in APC (Stettler and Axel, 2009; Isaacson, 2010; Davison and Ehlers, 2011; Wilson and Sullivan, 2011). Haberly proposes that APC is an analog of associative cortices more generally (Haberly, 2001). Mitral cell axons arrive in Layer Ia, making *en passant* contacts with pyramidal cell dendrites (Haberly, 2001; Isaacson, 2010; Wilson and Sullivan, 2011). Odor encoding is sustained by excitatory associational connections (Rennaker et al., 2007; Poo and Isaacson, 2011). Based on spines per dendrite in Layer Ia (Knafo et al., 2005), there is a relatively large “fan in” from mitral cells to single pyramidal cells. Such connectivity may implicate oscillations in odor decoding. In both OB and APC odor representation is best characterized as a dynamic spatiotemporal pattern (Laurent, 1996, 1997; Friedrich and Laurent, 2001; Rennaker et al., 2007; Restrepo et al., 2009).

Sparse representations as seen in OB and APC are proposed to provide a large repertoire of representations in restricted networks (Shadlen and Newsome, 1998; Olshausen and Field, 2004). Distributed representations confer the benefit of resistance to network degradation (Slotnick and Bisulco, 2003; Slotnick et al., 2004; Bracey et al., 2013).

## Learning and odor representations

Learning-related modulations of odor representations have been examined through imaging and electrophysiology. At the glomerular level olfactory learning enlarges learned-odor-associated glomeruli in rat pups (Woo et al., 1987), increases sensory neurons (Jones et al., 2008), increases sensory neuron output (Kass et al., 2013), and strengthens odor responses in weakly-activated glomeruli (Fletcher, 2012) in adult mice.

There are also increases in cFos labeled juxtaglomerular cells in rat pups (Woo and Leon, 1991). Zif268-labeled granule cells increase in signal strength, but not in overall numbers with appetitive conditioning in adult mice (Busto et al., 2009).

Increased synchrony of mitral cell spikes to learned odors also occurs in adult mice (Doucette et al., 2011). Rewarded vs. unrewarded odors had differing mitral firing rates, which reversed with reward reversal. The firing divergences were transient (Doucette and Restrepo, 2008).

Anterior piriform cortex pyramidal cell odor representations decrease after reward pairing in cFos images in adult rats (Roullet et al., 2005). In rat pups, cFos representations increased in APC with odor/shock pairing while granule cell odor representations in OB decreased (Roth and Sullivan, 2005; Roth et al., 2006). Using electrophysiology, Chapuis and Wilson (2012) demonstrated a decrease in correlated firing when rats discriminated highly similar odors and an increase in correlated firing when two odors were associated to reward. The minimal number of active APC neurons necessary for behavioral discrimination has been estimated at 300–500 (Choi et al., 2011).

## *Arc*-indexed odor representations and learning

In recent experiments we used two odor learning paradigms to investigate representation modifications: (1) early odor preference learning in rat pup (Figure 1B); and (2) go-no-go odor discrimination learning in adult rat (Figure 1C). We employed the cellular compartment analysis of temporal activity by fluorescence *in situ* hybridization (catFISH) technique to visualize *Arc* mRNA and characterize odor representations. *Arc*, as one of the immediate early genes, offers an important feature for examining representations repeatedly in the same animal (Guzowski et al., 2005). It is temporally relocated from the nucleus, where it appears 5 min after recruitment, to cytoplasm where it asymptotes ~25 min later. Thus cells where *Arc* transcription is recently recruited by odor can be compared to the cells that responded to the same or a different odor at an earlier time point (Shakhawat et al., 2014a,b).

### Rat pup odor preference learning

In the rat pup model (Shakhawat et al., 2014a; Figure 1B), ~7–8% of the mitral cells sampled responded to peppermint odor in the dorsolateral OB. Consistent with present models of OB odor representation this is a sparse representation. When a second presentation of peppermint was given to naïve animals the network activated was again ~7–8% of sampled neurons. The overlap i.e., the proportion of neurons responding to peppermint twice was surprisingly small. Thirty percent of the odor-activated neurons participated in both representations of peppermint. This result is at odds with the theoretical benefit of sparse representations, which as mentioned, are thought to maximize memory capacity. For that idea, it is usually assumed that each sparse representation gives a reliable encoding of any given memory.

What might be the source of this representational variability? Subtle contextual changes driven by top-down cortical inputs are a likely candidate (Restrepo et al., 2009). Thus one possibility is that there is additional information in each of these odor representations. For example, visual context is encoded even at the level of the OB (Mandairon et al., 2014).

Another utility of the observed variability may be noise modulation of signal processing. Noise in neuronal circuits enhances the discriminability of representations (Ermentrout et al., 2008). Evoked variability in each odor representation might increase the likelihood of a response in the APC and enhance discriminability.

The effects of reward on the peppermint representation were to increase the proportion of repeatedly activated neurons in that representation, but not to change its overall size (Shakhawat et al., 2014a). The proportion of neurons responding to both peppermint events increased to ~50%. An increase in the stable component of the peppermint representation would increase saliency and improve discriminability. Electrophysiology suggests a similar effect occurs in adult rodent OB: synchronous firing across mitral cells is enhanced by learning (Doucette et al., 2011).

While *Arc* is not normally recruited in interneurons (Vazdarjanova et al., 2006; McCurry et al., 2010), it is recruited in granule cells. Granule cell *Arc* revealed a similar pattern of effects to that of mitral cells (Shakhawat et al., 2014a). The peppermint representation indexed by granule cells was ~5% of the population sampled. This was unchanged by training. The granule cells responding to the same odor twice was ~25% of the representation and increased to ~50% with training. The *Arc* pattern for an unrewarded odor did not change for mitral or granule cells (Shakhawat et al., 2014a).

While mitral cells have not been imaged in earlier learning studies, granule cell representations in rat pups decreased with odor/shock pairings using cFos (Roth and Sullivan, 2005; Roth et al., 2006). The source of the discrepancy from the *Arc* findings is unclear. Stronger granule cell staining by Zif268, but no change in representation size, with appetitive training in adult mice (Busto et al., 2009) is consistent with the *Arc* results. The odor specific granule cell representation indexed by *Arc* also accords with theoretical predictions for the OB (Koulakov and Rinberg, 2011).

The modulation of granule cell ensembles by training is consistent with our understanding that granule cells partner with particular odor input representations and selectively modulate or track changes in those representations (Migliore et al., 2007; Yu et al., 2014). It is assumed generally that inhibition increases in concert with excitation in neural circuits (Vogels et al., 2011). Comparable increases in the stable proportion of the mitral and granule cell representations of rewarded odor are consistent with this assumption.

In the same rat pups, APC representations paralleled what was seen in the OB (Shakhawat et al., 2014a). The pyramidal cell representation was sparser with only ~1% of neurons of those sampled participating. This contrasts with a peppermint representation size in adults of about 3–5% (Shakhawat et al., 2014b). However, only one-third of mitral cells axons are mature at this age. The smaller representation likely reflects reduced APC input (Sarma et al., 2011). The stable component of the representations for repeated odors without learning was ~20%. Given a 1% representation size, one would estimate ~1500 pyramidal neurons for the odor representation (~150,000 total in one APC, Capurso et al., 1997; Duffell et al., 2000), with a stable component of only ~300 neurons, at the limit of the predicted population needed for discrimination (Choi et al., 2011). With odor preference training, this proportion increased to ~40% with no change in representation size. Representation components were unchanged without paired reward (Shakhawat et al., 2014a). The similarity of modifications in OB and APC representations are likely supported by their mutual connectivity (Restrepo et al., 2009; Boyd et al., 2013), but studies have shown that each structure can modify connectivity when reward and odor signals are paired (Sullivan et al., 2000; Lethbridge et al., 2012; Morrison et al., 2013; see also Thum et al., 2007).

### Adult rat go-no-go odor discrimination learning and anterior piriform cortex

Adult rats were tested in three types of odor discrimination (Shakhawat et al., 2014b). The first was a simple discrimination of two dissimilar odors, peppermint (rewarded) vs. vanillin (no reward; Figure 1C1); the second involved a mixture of the two odors, which were rewarded while a third odor was not (Figure 1C2). The third discrimination employed two highly similar mixtures initially difficult to discriminate (Figure 1C3).

Electrophysiology had not revealed learning differences in APC firing patterns for simple discriminations (Chapuis and Wilson, 2012). But with *Arc*, it was possible to observe that representation size became significantly smaller in trained rats (2.5%) vs. untrained rats (5%), with a reduction in the variable neuronal component. The overlap component did not show an absolute increase with training, but became a larger proportion of the representation. Thus, as in the rat pups (Shakhawat et al., 2014a), the stable component of the APC odor representation increased with learning from 25% to 40% (Shakhawat et al., 2014b). A learning-induced decrease in APC odor representation size was previously seen with cFos (Roullet et al., 2005).

In the mixture discrimination, the two individual odor components were responded to as rewarded odors during behavioral probes, suggesting rats learned the components during mixture training or pattern-completed from partial cues. The degree of overlap between peppermint and vanillin in control rats was ~20% while overlap was ~45% in trained rats, consistent with the two odors becoming representationally highly similar (Shakhawat et al., 2014b).

In the difficult discrimination, the odor representation size was ~3% and did not change with learning (Shakhawat et al., 2014b). The proportion of cells responding to the similar odors was 23%, not different from the overlap seen to repeated odors (25–30%). After training to differentiate the odors, the proportion of cells responding twice to the two similar odors (overlap) was significantly less (~13%). The mechanisms by which reward-mediated decorrelation is achieved are not well understood. Cholinergic inputs have been shown to modulate odor pattern separation in the APC (Chapuis and Wilson, 2013).

### General inferences regarding reward effects on odor representations

The mechanisms for associating odor and reward, presumably resulting in the increases in stability components seen here, have been examined earlier. In the rat pup, norepinephrine paired with odor can alter behavior and AMPA receptor-mediated connectivity in OB (Cui et al., 2011; Yuan and Harley, 2012) and APC (Fontaine et al., 2013; Morrison et al., 2013). But the common assumption that spike timing-dependent plasticity in cortical circuits underlies representational changes has not been supported when examining optimal temporal relationships between odor input and reward signals using recording of cell firing to define the two inputs (Ito et al., 2008). It has subsequently been recognized that a neuromodulator is required as a third partner to enable spike timing dependent plasticity (STDP; Pawlak et al., 2010; Cassenaer and Laurent, 2012). This appears likely to occur in rat pup odor learning. We suggest that priming of co-incident odor-activated pre- and post-synaptic elements in OB and APC of rat pups, which are then strengthened by norepinephrine, is the most probable mechanism of the representation modifications seen in pups.

### Novel representational features indexed by *Arc*

The two novel results that *Arc* imaging made possible was the finding of high variability in repeated odor representations and the finding of a high degree of overlap between representations of distinct odors. If cells encoding distinct odors were drawn at random from populations with replacement such that the same cell could participate in multiple representations, then the predicted overlap would be the representational size squared. In *Arc* studies, representational size varies from 1% to 10%, consistent with other estimates (e.g., Lin da et al., 2006, OB 3–10%; Stettler and Axel, 2009, APC 3–15% at low concentrations), and the predicted overlap would be no more than 1%. The overlap observed for the odors peppermint and vanillin, which according to the earlier spatial maps are represented differentially in OB (see Johnson and Leon’s 2-DG odor glomerular maps^1^), was ~20% in OB and APC. What is the source of this large overlap? The discovery of primed or widely responsive neuron subsets comprising about 20–25% of studied populations in a variety of cortical circuits including APC (Yassin et al., 2010; Zhan and Luo, 2010; Luczak and Maclean, 2012; Mizuseki and Buzsaki, 2013; Reuveni et al., 2013), may account in part for larger overlap. Another contributing factor might be that the two odors share molecular features which, despite being distributed, would still result in representations with overlapping neurons (Saito et al., 2009; Ma et al., 2012). A greater diversity of odors needs to be tested with *Arc*.

### *Arc*’s relation to odor evoked activity

Finally we come to the issue of what *Arc* itself indicates. While it is related to neuronal activation, neural firing cannot be its sole initiator. Mitral cells fire at high spontaneous levels (Rinberg et al., 2006). This activity may be driven by muscarinic inputs in conjunction with the intrinsic variable biophysical properties of mitral cells (Padmanabhan and Urban, 2010; Angelo and Margrie, 2011) and for that reason does not recruit *Arc* activation. *Arc* recruitment is likely linked to glutamatergic excitatory synaptic driving (Cole et al., 1989), as occurs with odor input.

But even if that is the case, one must ask which driving patterns are encoded by *Arc* recruitment. Odor activation firing patterns in OB and APC are dynamic spatially and temporally (Friedrich and Laurent, 2001; Rennaker et al., 2007; Schaefer and Margrie, 2007), although encoding and perception of odors can occur very rapidly (Uchida and Mainen, 2003; Wesson et al., 2008). At different time points different cells fire and these responses are embedded in the high spontaneous background. Static odor representations have been extracted statistically at different points and appear to synchronize with sniffs (~250 ms). While a single sniff suffices for odor discrimination, the odor representation continues to evolve (Patterson et al., 2013). In subsequent breaths the statistical odor template changes and stabilizes (see also Broome et al., 2006). Does *Arc* represent the initial pattern and its variability or the combined early and later patterns and their cumulative variability? This would be another contribution to the high variability observed.

How many neurons are excited in a single synchronized odor representation? The size of odor representations appears to be normalized and varies less for concentration increases (Cleland, 2010) than one might expect given the wider glomerular activation as concentration increases (Meister and Bonhoeffer, 2001). Increased concentrations are easier to discriminate (Escanilla et al., 2008) and this may relate to stronger peak excitation (Cleland, 2010); latency for initial spikes is also reduced (Stopfer et al., 2003), suggesting an importance of early timing. From *Arc* patterns, we would predict a larger stable component with increasing concentrations supporting more rapid discriminations.

## Conclusion

*Arc*-indexed cell networks portray odor representations in both OB and APC as sparse and distributed consistent with current understanding. *Arc*-indexed networks also reveal a considerable variability in the awake mammalian odor representation consistent with the electrophysiological evidence. This methodology further reveals a larger component of common neuronal activation for distinct odors than predicted by theory. Appetitive learning modifies odor representations to increase the proportion of stable neurons. Network representations can also decrease the proportion of stable neurons when increased behavioral discrimination is required.

## Conflict of interest statement

The authors declare that the research was conducted in the absence of any commercial or financial relationships that could be construed as a potential conflict of interest.

## Footnotes

^1^http://gara.bio.uci.edu/
